# Impacts of prescribed burning on *Sphagnum* mosses in a long-term peatland field experiment

**DOI:** 10.1371/journal.pone.0206320

**Published:** 2018-11-01

**Authors:** Alice Noble, John O’Reilly, David J. Glaves, Alistair Crowle, Sheila M. Palmer, Joseph Holden

**Affiliations:** 1 water@leeds, School of Geography, University of Leeds, Leeds, United Kingdom; 2 Ptyxis Ecology, Lambley, Brampton, United Kingdom; 3 Natural England, Foss House, Kings Pool, Peasholme Green, York, United Kingdom; Helmholtz Centre for Environmental Research - UFZ, GERMANY

## Abstract

Understanding fire impacts on peatland vegetation can inform management to support function and prevent degradation of these important ecosystems. However, time since burn, interval between burns and number of past burns all have the potential to modify impacts. Grazing regime may also affect vegetation directly or via an interaction with burning. We used new, comprehensive survey data from a hillslope-scale field experiment initiated in 1954 to investigate the effects of burning and grazing treatments on *Sphagnum*. Historical data were consulted to aid interpretation of the results. The unburned reference and the most frequently burned (10-year rotation) treatments had greater *Sphagnum* abundance and hummock height than intermediate treatments (20-year rotation and no-burn since 1954). Abundance of the most common individual species (*S*. *capillifolium*, *S*. *subnitens* and *S*. *papillosum*) followed similar patterns. Light grazing had no impact on *Sphagnum*-related variables, nor did it interact with the burning treatments.These results suggest that in some cases fire has a negative impact on *Sphagnum*, and this can persist for several decades. However, fire return interval and other factors such as atmospheric pollution may alter effects, and in some cases *Sphagnum* abundance may recover. Fire severity and site specific conditions may also influence effects, so we advise consideration of these factors, and caution when using fire as a management tool on peatlands where *Sphagnum* is considered desirable.

## 1. Introduction

Peatlands, which cover around 4.23 million km^2^ globally [1], are important landscapes for biodiversity, carbon storage and hydrological functions [2]. On many peatlands, particularly at high latitudes, *Sphagnum* mosses are central to ecosystem function, influencing hydrology [3], chemistry [4], temperature [5] and microtopography [6], as well as sequestering carbon [7]. Fire is common on peatlands worldwide and includes both wildfire and prescribed burning for purposes including wildfire prevention, land clearance, agricultural grazing and game management [8–10]. Knowledge of the impacts of fire on *Sphagnum* is therefore vital to inform fire-impact predictions and nature conservation management decisions.

Prescribed burning occurs in many peatland ecosystems worldwide including areas of North America [11] and Europe [12, 13]. Fires are often controlled to burn vegetation without igniting the underlying peat, so results from studies of wildfire, where moss and surface peat layers can be consumed, may not be directly applicable. In the UK, prescribed burning is commonly carried out on patches of up to c.4000 m^*2*^ (0.4ha) in rotations of around 8–25 years. The canopy layer, which on UK peatlands is usually dominated by dwarf shrubs (including *Calluna vulgaris*) and sedges (commonly *Eriophorum vaginatum* and *E*. *angustifolium*) is burned to create a range of vegetation ages suitable for nesting and foraging of the game bird red grouse (*Lagopus lagopus scotica*). Official guidance advocates a strong presumption against burning on deep peat [14], but there is evidence that burning has increased on UK peatlands in recent decades [9, 15, 16].

While national-scale work has shown that there is less *Sphagnum* cover on peatlands subject to prescribed burning in England [17], results from local and regional scale studies suggest that effects can vary depending on fire severity and return interval [18, 19]. Burning may influence *Sphagnum* by heat damage or combustion with varying recovery prospects [18, 20, 21], and changes in substrate properties can also have an effect. For example, higher near-surface peat bulk densities and lower soil water availability on recently burned sites [22] can limit *Sphagnum* growth [23], and more extreme peat surface temperatures in the years after burning [24] may also have a negative impact [25]. Ash deposition from burning may cause short term cation enrichment [26] with potentially positive effects for some *Sphagnum* species [23], but this may also increase competition, and some cations may be depleted in the longer term (2+ years after burning) [27]. Previous field studies have reported *Sphagnum* abundance [17, 19], but hummock height, which may also be affected by burning representing a change in biomass, is seldom reported. Understanding burning effects on both abundance and hummock height would contribute to a more complete knowledge of impacts on carbon sequestration and other ecosystem services.

The Hard Hill vegetation burning and grazing experiment at Moor House National Nature Reserve in the North Pennines, UK was established in 1954. The main experiment includes three burning treatments with plots burned on either short (10-year) or long (20-year) rotations, or burned once in 1954/55 and left unburned since (S, L and N plots; Table 1). Reference (R) plots were established adjacent to the main experiment plots, outside of the 1954 burn area. Studies of the vegetation of the main experiment plots were published in the 1970s [28] and 1980s [29]. More recent work has shown greater *Sphagnum* abundance [19] and lower *Sphagnum* propagule availability [30] on the 10-year (S) rotation plots compared to 20-year (L) and no-burn since 1954 (N) plots. However, a direct comparison of *Sphagnum* abundance between the experiment and reference (R) plots has not previously been carried out, so it is not known how the experimental treatments compare to surrounding vegetation. Furthermore, previous surveys may not have captured rarer or less evenly distributed *Sphagnum* species [31].

**Table 1:** Burning treatments in the Hard Hill experiment.

Code	Treatment
R	Reference, unburned for 90+ years
N	No-burn since 1954/55, unburned for 60+ years
L	Long (20-year) rotation
S	Short (10-year) rotation

Here we present the results of a comprehensive survey of the main experiment and reference plots, including hummock height data and mapping of all *Sphagnum* patches in every plot at species level, with the aim of investigating the effect of burning treatments. This represents the most complete survey of *Sphagnum* in a burning experiment to date and the first time the Hard Hill experimental treatments have been compared to a reference. Changes over time are also considered with reference to data from past surveys. The results are discussed in the context of the potential processes responsible for burning impacts on *Sphagnum* and implications for future burn management and policy.

## 2. Methods

### 2.1 Experimental design

**Fig 1:**
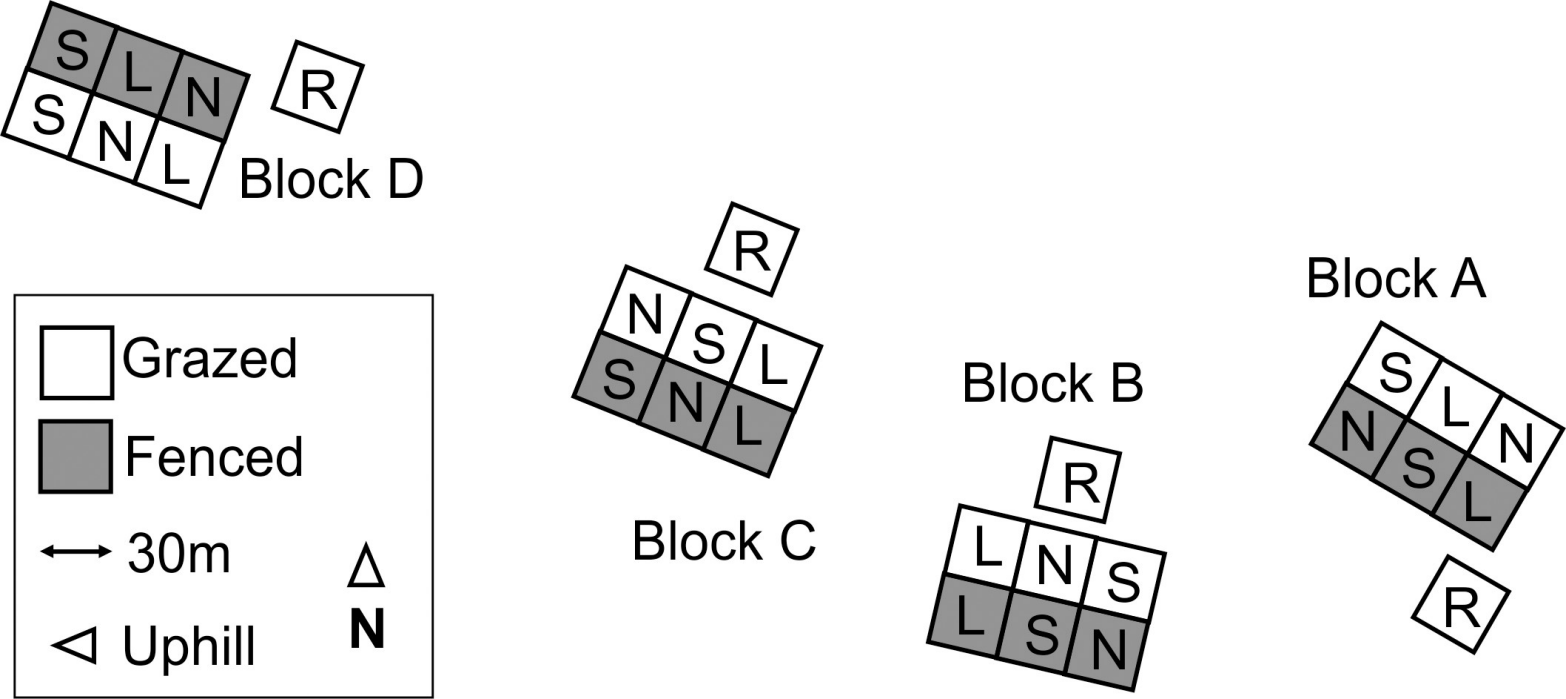
Layout of the Hard Hill experimental plots (R = reference, N = no-burn since 1954, L = long (20-year) rotation, S = short (10-year) rotation.

The Hard Hill experiment consists of four 90m x 60m blocks, each made up of six 30m x 30m plots. At the start of the experiment in 1954, half of each block (three plots) was fenced to exclude grazing, and within each half three burning treatments (S, L and N) were allocated at random. All of the main experiment plots were burned at the start of the experiment and the S and L plots have been burned on approximately 10- and 20-year rotations respectively since then (subject to suitable weather conditions). Unfenced reference plots which had remained unburned for at least 30 years prior to 1954 [28] were established alongside each block outside of the initial burn areas (Fig 1). The burning and survey schedule of the experiment is described in Lee et al. [19], which also provides information on the overall vegetation composition. Briefly, the plots comprise *Calluna vulgaris- Eriophorum vaginatum* blanket mire which is characteristic of much of the blanket bog in the English Pennines which has been modified to a greater or lesser extent by grazing and burning management and other impacts.

### 2.2 *Sphagnum* surveys

The 24 main experiment plots and four reference plots were surveyed between August 2015 and April 2016. Within each plot 10 transects were laid out at evenly spaced intervals. Transects were located at least 1.5 m away from the plot edges to avoid heavily trampled areas and edge effects and hence were between 22.5 and 27 m long. Survey data were recorded at 10 evenly spaced pin points along each transect (100 points total per plot). *Sphagnum* presence or absence was recorded at each pin point, and where *Sphagnum* was present the species was identified. *S*. *capillifolium* ssp. *capillifolium* (Ehrh.) Hedw. and *S*. *capillifolium* ssp. *rubellum* (Wilson) M.O.Hill were initially recorded separately, but the two subspecies could not always be differentiated with certainty so analysis was undertaken at species level. *Sphagnum* patch length and width were measured to the nearest cm at the widest points parallel and perpendicular to the transect, and patch area was calculated using the formula $area=\pi \;{\left(\frac{length+width}{4}\right)}^{2}$. *Sphagnum* patch height was measured to the nearest cm by inserting a cane vertically into the patch at the pin point until it met resistance from the underlying peat. For the 24 main experiment plots the height data were recorded approximately 6 months later than the frequency, species identity and length/width data, so it was not possible to replicate precisely the original pin points.

Alongside the transect survey, a mapping survey was conducted to record the location, species and approximate area of every *Sphagnum* patch in all 24 plots. This was carried out by walking along the nine 2.5–3 m wide strips between transects in each plot and drawing each *Sphagnum* patch encountered as a polygon on a corresponding map. Transects were marked with tape measures to provide a reference for patch position. To calculate *Sphagnum* frequency from the resulting maps, a transparent overlay with 1296 regular grid squares for each plot was used. The number of squares partly or entirely occupied was counted for each *Sphagnum* species, and *Sphagnum* as a genus.

### 2.3 Past surveys

Vegetation surveys which recorded *Sphagnum* and other species in some or all of the Hard Hill plots were carried out in 1961, 1965, 1972/3, 1982, 1991, 2001 and 2011 using various recording methods (Table 2). Analysis of the 1972–2001 data from the S, L and N plots and investigation of change between 1965 and 2011 in the R plots was carried out by Lee et al. [19]. Rawes and Hobbs [28] presented results from the 1961 survey, but differences in *Sphagnum* between treatments were not discussed. No comparison of the N and R plot data from the 1965 data has been published to date. Therefore, to support the interpretation of the 2015/16 data, we analysed *Sphagnum* abundance from the plots surveyed in 1961 (all main experiment plots) and 1965 (grazed N and R plots).

**Table 2:** Summary of past surveys of Hard Hill plots. Burn treatments are short rotation (S), long rotation (L), no-burn since 1954 (N) and unburned reference (R).

Year(s)	Treatments	Survey type	Samples per plot
1961	S, L, N	Domin	25 x 1 m^2^ quadrats
1965	N, R	Domin	5 (R) or 10 (N) 1 m^2^ quadrats
1972/3, 1982, 1991, 2001	S, L, N	Point quadrats	20 x 1 m^2^ quadrats x 5 pins
2011	R	Domin	25 quadrats
2015/6	S, L, N, R	Transect/mapping	10 transects, 100 pin points

### 2.4 Data analysis

All statistical analyses were carried out using R 3.1.0 [32]. Data from the main experiment plots were analysed using split plot ANOVA with split plot nested within block as the error term and burning, grazing and their interaction as factors. Second, data from the grazed main experiment plots and reference plots were compared using ANOVA with burn status and block as factors. The two types of analysis both represent balanced experimental designs, and were carried out separately to account for the absence of a fenced reference treatment.

Dependent variables in the 2015–2016 data included transect hits, patch size, hummock height, and proportion of map squares occupied for *Sphagnum* as a genus. The proportion of transect hits and map squares occupied were calculated for individual species and where species were present in more than one percent of samples these variables were analysed in the same way.

Analysis of the 1961 and 1965 historical data used similar methods, with split plot ANOVA for the 1961 main experiment data and ANOVA with burn status and block as factors for the 1965 N and R plot data. Domin scores were transformed using the Domin 2.4 transformation (Currall 1987) to give an approximation of percentage cover. To account for potential effects of unequal sampling effort between treatments only the first five quadrats from each grazed N plot in 1965 were used in the analysis.

For each ANOVA model homogeneity of variances and normality of residuals were inspected graphically, and where appropriate data were transformed to reduce skew and/or heteroscedasticity. Tukey’s Honest Significant Difference (HSD) pairwise test was used to investigate differences between treatment combinations.

## 3. Results

### 3.1 Comparison of main experiment plots in 2015–16

**Fig 2:**
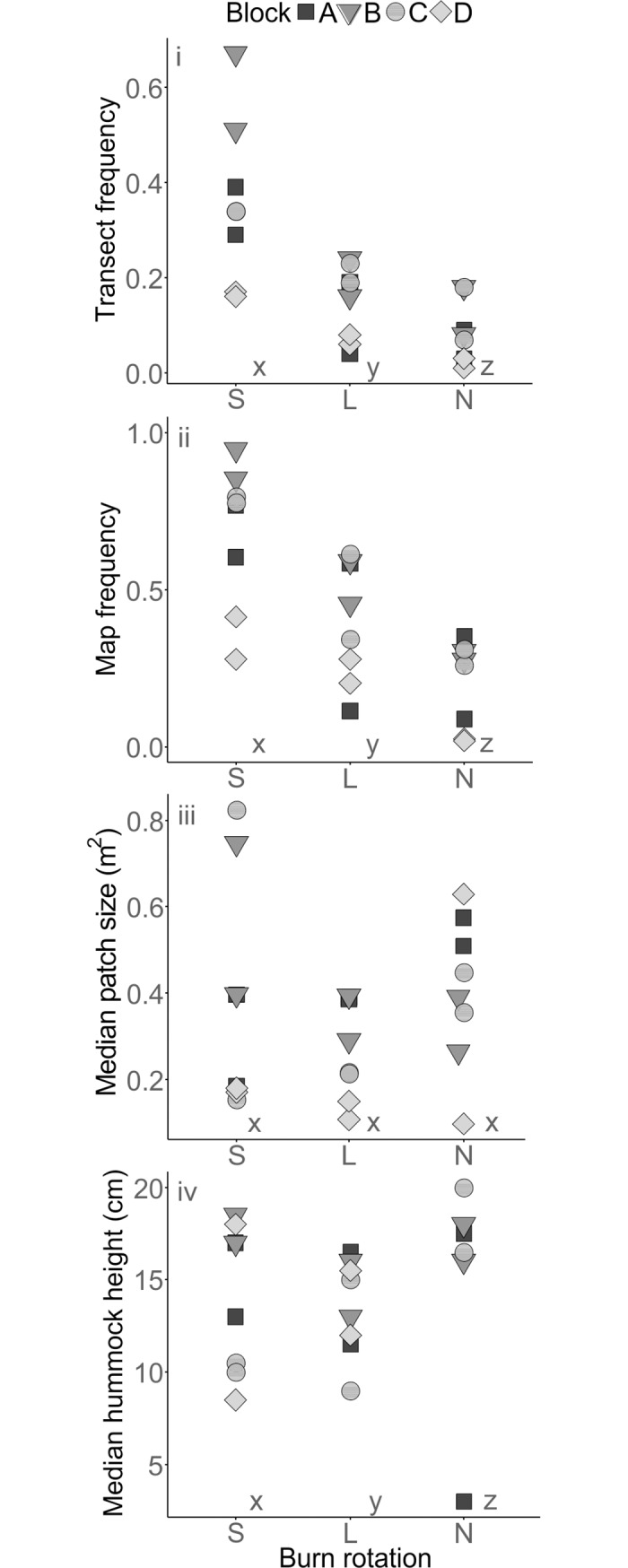
Values for i) transect frequency (0–1), ii) map frequency (0–1), iii) median patch size (m^2^) and iv) median hummock height (cm) of *Sphagnum* for all plots (grazed and fenced) within the main experiment in 2015–16. Burn treatments are short rotation (S), long rotation (L) and no-burn (N). Treatments which do not share an x, y, z letter coding are significantly different (p < 0.05) according to Tukey HSD tests.

For *Sphagnum* spp. (hereafter *Sphagnum*), both transect hits and map frequency were significantly associated with burn treatment (Table 3), with more frequent occurrence in S plots than in L and N plots and more frequently in L plots than in N plots (Fig 2). However, neither median patch area nor hummock height were significantly associated with burn status (Table 3). The only species present at more than 1% of transect pin points was *S*. *capillifolium*, which was also significantly associated with burn treatment (Table 3), occurring more frequently in S plots than in L and N plots. *S*. *capillifolium*, *S*. *subnitens* and *S*. *papillosum* all occurred in more than 1% of the map squares and all three were significantly associated with burn treatment (Table 3). *S*. *capillifolium* occurred more frequently in S plots than in L and N plots and more frequently in L plots than in N plots, while *S*. *subnitens* and *S*. *papillosum* occurred more frequently in S plots than in N plots. Neither grazing treatment nor its interaction with burn treatment were significantly associated with any of the *Sphagnum*-related variables tested.

**Table 3:** Results of split plot ANOVAs for *Sphagnum*-related variables (transformations in brackets) recorded in the main experiment plots (grazed and fenced S, L and N) in 2015–16.

Dependent variable	Error	Factor	Df	Sum sq	Mean sq	F value	Pr(>F)
*Sphagnum* spp.	Block	Residuals	3	0.2161	0.0720		
Transect frequency	Grazing	Grazing	1	0.0005	0.0005	0.04	0.861
(square root)		Residuals	3	0.0396	0.0132		
	Within	Burn	2	0.4167	0.2084	48.07	<0.001
		Grazing:Burn	2	0.0152	0.0076	1.75	0.215
		Residuals	12	0.0520	0.0043		
*Sphagnum* spp.	Block	Residuals	3	0.4740	0.1580		
Map frequency	Grazing	Grazing	1	0.0312	0.0312	0.78	0.443
		Residuals	3	0.1209	0.0403		
	Within	Burn	2	0.9110	0.4555	45.25	<0.001
		Grazing:Burn	2	0.0111	0.0055	0.55	0.591
		Residuals	12	0.1208	0.0101		
*Sphagnum* spp.	Block	Residuals	3	0.1440	0.0480		
Patch area (m^2^)	Grazing	Grazing	1	0.0115	0.0115	0.24	0.656
		Residuals	3	0.1426	0.0475		
	Within	Burn	2	0.0886	0.0443	1.29	0.310
		Grazing:Burn	2	0.0985	0.0493	1.44	0.276
		Residuals	12	0.4113	0.0343		
*Sphagnum* spp.	Block	Residuals	3	40.37	13.46		
Hummock height (cm)	Grazing	Grazing	1	19.26	19.26	0.57	0.505
		Residuals	3	101.54	33.85		
	Within	Burn	2	9.33	4.66	0.31	0.702
		Grazing:Burn	2	36.58	18.29	1.20	0.340
		Residuals	10	151.92	15.19		
*S*. *capillifolium*	Block	Residuals	3	0.2348	0.0783		
Transect frequency	Grazing	Grazing	1	0.0032	0.0032	0.27	0.640
(square root)		Residuals	3	0.0358	0.0119		
	Within	Burn	2	0.3408	0.1704	37.29	<0.001
		Grazing:Burn	2	0.0251	0.0126	2.75	0.104
		Residuals	12	0.0548	0.0046		
*S*. *capillifolium*	Block	Residuals	3	0.4931	0.1644		
Map frequency	Grazing	Grazing	1	0.0359	0.0359	1.07	0.376
(square root)		Residuals	3	0.1003	0.0334		
	Within	Burn	2	0.5407	0.2703	46.26	<0.001
		Grazing:Burn	2	0.0042	0.0021	0.36	0.704
		Residuals	12	0.0701	0.0058		
*S*. *subnitens*	Block	Residuals	3	0.0148	0.0049		
Map frequency	Grazing	Grazing	1	0.0018	0.0018	0.34	0.602
(square root)		Residuals	3	0.0164	0.0055		
	Within	Burn	2	0.1027	0.0513	8.29	0.005
		Grazing:Burn	2	0.0013	0.0007	0.11	0.898
		Residuals	12	0.0743	0.0062		
*S*. *papillosum*	Block	Residuals	3	0.0089	0.0030		
Map frequency	Grazing	Grazing	1	0.0036	0.0036	0.62	0.487
(square root)		Residuals	3	0.0173	0.0058		
	Within	Burn	2	0.0767	0.0384	9.56	0.003
		Grazing:Burn	2	0.0040	0.0020	0.50	0.616
		Residuals	12	0.0481	0.0040		

### 3.2 Comparison of reference and grazed main experiment plots in 2015–16

**Fig 3:**
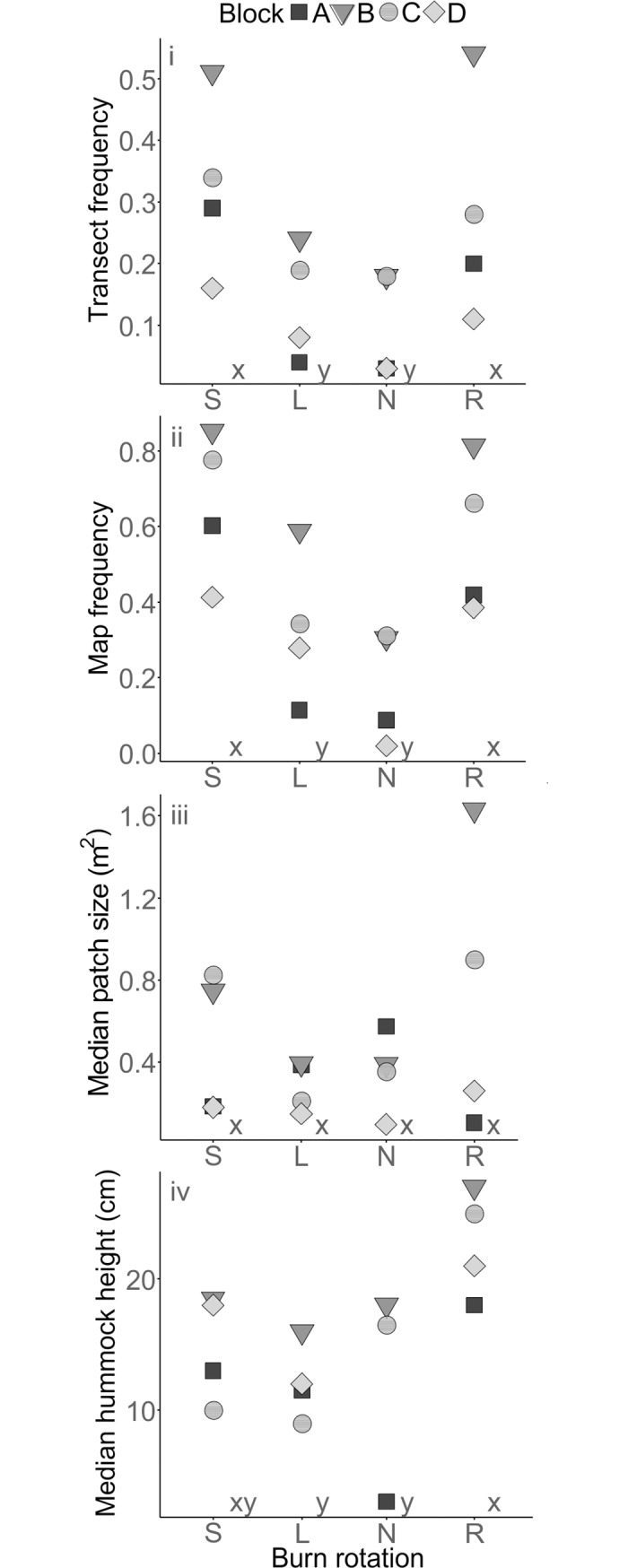
Values for i) transect frequency (0–1), ii) map frequency (0–1), iii) median patch size (m^2^) and iv) median hummock height (cm) of *Sphagnum* for grazed plots within the main experiment, and reference plots in 2015–16. Burn treatments are short rotation (S), long rotation (L), no-burn (N) and reference (R). Treatments which do not share an x, y, z letter coding are significantly different (p < 0.05) according to Tukey HSD tests.

*Sphagnum* was significantly associated with burn status (Table 4), occurring more frequently in R and S plots than in L and N plots according to both the transect and map data (Fig 3). Patch area was not significantly associated with burning status, but hummock height was (Table 4), with higher values in R plots than in L or N plots (Fig 3). In both the transect and map data, *S*. *capillifolium* occurred more frequently in R and S plots than in L and N plots (Table 4). *S*. *subnitens* and *S*. *papillosum* were both significantly associated with burning status in the map data (Table 4) and were more frequent in S than in N plots, with *S*. *papillosum* also more frequent in S than in R plots.

**Table 4:** Results of ANOVAs for *Sphagnum*-related variables recorded in the 2015–16 survey in the grazed main experiment (S, L and N) and reference (R) plots. Transformations of the dependent variables are noted in brackets.

Spp.	Dependent variable	Factor	Df	Sum sq	Mean sq	F value	Pr(>F)
*Sphagnum* spp.	Transect frequency	Burn	3	0.1389	0.0463	11.24	0.002
		Block	3	0.1772	0.0591		
		Residuals	9	0.0371	0.0041		
	Map frequency	Burn	3	0.5790	0.1930	32.17	<0.001
		Block	3	0.3696	0.1232		
		Residuals	9	0.0540	0.0060		
	Patch area (m^2^)	Burn	3	0.4467	0.1489	1.25	0.348
		Block	3	0.9077	0.3026		
		Residuals	9	1.0705	0.1190		
	Hummock height (cm)	Burn	3	283.30	94.44	6.58	0.015
		Block	3	145.90	48.62		
		Residuals	8	114.80	14.35		
S. *capillifolium*	Transect frequency	Burn	3	0.1012	0.0337	8.94	0.005
		Block	3	0.1857	0.0619		
		Residuals	9	0.0340	0.0038		
	Map frequency	Burn	3	0.3693	0.1231	23.04	<0.001
	(square root)	Block	3	0.3555	0.1185		
		Residuals	9	0.0481	0.0053		
S. *subnitens*	Map frequency	Burn	3	0.3693	0.1231	23.04	<0.001
	(square root)	Block	3	0.3555	0.1185		
		Residuals	9	0.0481	0.0053		
S. *papillosum*	Map frequency	Burn	3	0.0713	0.0238	5.42	0.021
	(square root)	Block	3	0.0136	0.0045		
		Residuals	9	0.0395	0.0044		

### 3.3 Past surveys

**Fig 4:**
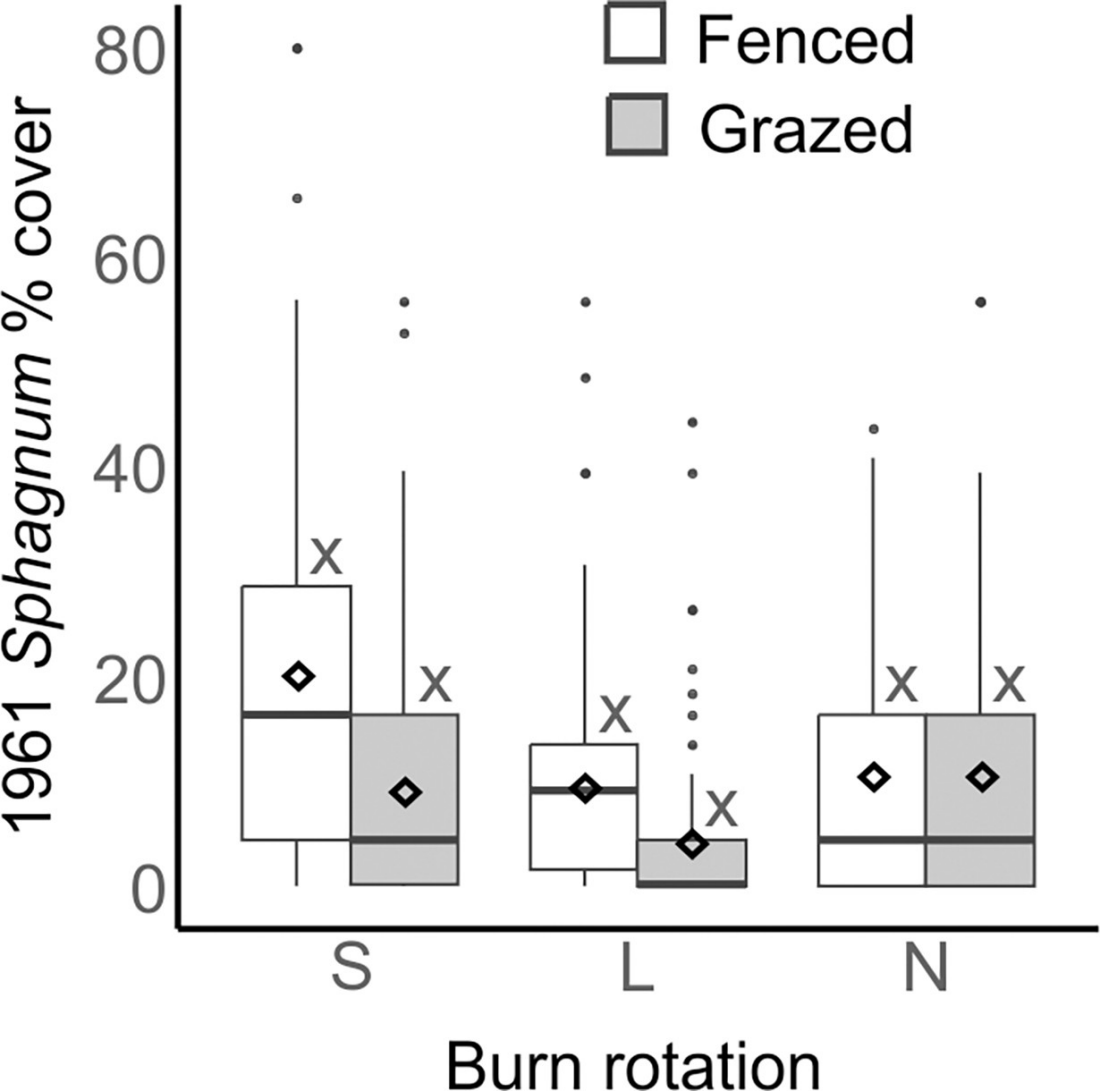
Boxplots showing Sphagnum % cover (transformed from domin scale values) in grazed and fenced plots within the main experiment in 1961 (data from 25 quadrats in each of 4 plots per treatment; n = 100). Burn treatments are short rotation (S), long rotation (L) and no-burn (N). The horizontal line, box, whiskers, dots and ◊ indicate the median, upper and lower quartiles, minimum and maximum excluding outliers, outliers and mean respectively. Treatments sharing the letter x coding are not significantly different (p > 0.05) according to Tukey HSD tests.

**Fig 5:**
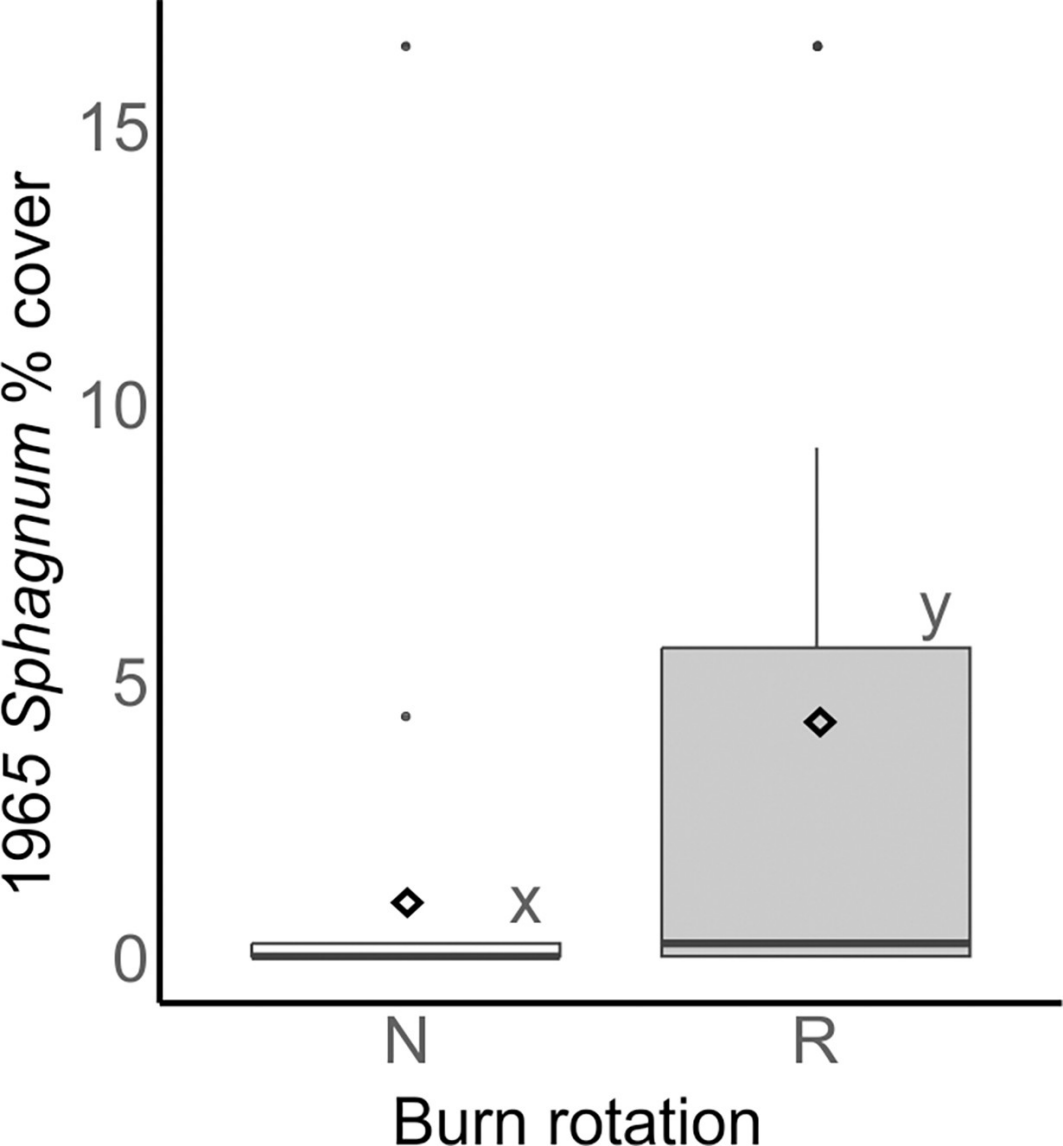
Boxplots showing *Sphagnum* cover (recorded on the domin scale and transformed to %) in reference (R) and grazed no-burn (N) plots (data from 5 quadrats in each of 4 plots per treatment; n = 20). The horizontal line, box, whiskers, dots and ◊ indicate the median, upper and lower quartiles, minimum and maximum excluding outliers, outliers and mean respectively. Treatments which do not share an x, y, z letter coding are significantly different (p < 0.05).

Analysis of the 1961 data from the main experiment plots showed no significant difference in cover of *Sphagnum* according to burn treatment, grazing or their interaction (Table 5, Fig 4) seven years after the initial burn. Analysis of the data from N and R plots in 1965 found that the reference plots had significantly greater *Sphagnum* cover (Table 6, Fig 5).

**Table 5:** Results of ANOVA for *Sphagnum* cover (%; square root transformed) in the main experiment plots (grazed and fenced S, L and N) in 1961.

Error	Factor	Df	Sum sq	Mean sq	F value	Pr(>F)
Block	Residuals	3	258.9	86.29		
Block:Grazing	Grazing	1	136.19	136.19	5.28	0.105
	Residuals	3	77.43	25.81		
Block:Grazing:Burn	Burn	2	130.77	65.38	2.85	0.097
	Grazing:Burn	2	54.63	27.31	1.19	0.338
	Residuals	12	275.25	22.94		
Within	Residuals	576	1845	3.203		

**Table 6:** Results of ANOVA for *Sphagnum* cover (%; square root transformed) in the grazed no-burn (N) plots and reference (R) plots in 1965.

Factor	Df	Sum sq	Mean sq	F value	Pr(>F)
Burn	1	9.09	9.09	7.63	0.009
Block	3	20.04	6.68		
Residuals	35	41.72	1.19		

## 4. Discussion

### 4.1 Recording methods and *Sphagnum* occurrence

The mapping survey provided the most comprehensive measure of *Sphagnum* frequency, with a greater number of species recorded than in the transect survey. *Sphagnum* frequency recorded in the transect survey was systematically lower than in the map survey, but the pattern of results was similar (Table A in S1 Supporting Information, Figs 2 and 3). This indicates that on the scale of this experiment, transect sampling is an acceptable way to evaluate treatment effects on more common species, but mapping surveys may be a more appropriate method to record less frequent species.

*Sphagnum* as a genus, and the individual species which were common enough to analyse separately, appeared to respond to burning treatments similarly. However, it is possible that some of the species occurring in less than one percent of plots, which were not analysed separately, responded differently. For example, *S*. *angustifolium* did not occur in the main experiment plots but was the second most common species in the reference plots, and conversely *S*. *russowii* occurred only in the main experiment plots (Table A in S1 Supporting Information), but the relative rarity of these species within the Hard Hill plots means that it is difficult to confidently attribute these differences to burning effects. A greater number of species occurred in the 24 main experiment plots compared to the four reference plots (Table A in S1 Supporting Information), which was expected due to the greater area covered.

### 4.2 *Sphagnum* frequency in the main experiment plots

The results of the 2015–16 survey indicate that *Sphagnum* is most frequent in S plots (10-year rotation), followed by L plots (20-year rotation), and least frequent in the N plots (unburned since 1954). Previous work by Lee et al. [19] using data from point quadrat surveys in 1972/3, 1982, 1991, and 2001 showed that *Sphagnum* abundance was greatest in S plots, but did not report any significant difference between N and L plots. We found no significant differences in *Sphagnum* patch area or hummock height between treatments, which suggests that the difference in frequency could be due to more numerous patches in the more frequently burned treatments. The cause of this difference could be a more open canopy or increased bare ground after burning providing a release from competition and an opportunity for *Sphagnum* to establish [19]. Alternatively, ash produced by fire can release limiting nutrients such as phosphorus, which can promote moss spore germination [33] and growth of some *Sphagnum* species [23]. As the S plots have been burned most frequently (five times since 1954 compared to twice in L plots), there have been more potential establishment opportunities in this treatment. Furthermore, the shorter rotation in S compared to L plots means less biomass accumulation between burns [34] and therefore less fuel, potentially resulting in lower fire temperatures [35]. This may have reduced the chance of heat-related damage to existing *Sphagnum* [20], contributing to the greater abundance than in the L treatment.

It is also possible that atmospheric pollution at the time of burning is relevant. After the UK clean air act of 1956, levels of sulphur pollutants peaked around 1960 and subsequently declined [36–38]. High atmospheric pollution levels in the 1950s may therefore have inhibited *Sphagnum* regeneration after burning, resulting in vegetation dominated by other species, as observed in N plots which were last burned in 1954. However, in S and L plots, subsequent burns which occurred under reduced atmospheric pollution levels may have facilitated *Sphagnum* growth by reducing competition as discussed above. Though different sampling methods were used, the data from the 2015 transect survey and the point quadrats used by Lee et al. [19] both provide an estimate of percentage cover and comparison suggests that this may have increased recently, e.g., from 7% in the S grazed treatment in 2001 [19] to 33% in 2015. *Sphagnum* is known to be affected by atmospheric pollutants [39, 40] and Noble et al. [17] observed that an interaction between burning and atmospheric pollution was associated with *Sphagnum* abundance. Further study could help to clarify the processes behind such interactions.

Grazing treatment had no impact on any of the *Sphagnum*-related variables. Similarly Lee et al. [19] found no effect of burning on *Sphagnum* or overall vegetation composition, which they suggested may be due to the low density, summer only grazing regime at Hard Hill. Past work has found some evidence of higher density grazing impacting *Sphagnum* [28], and Noble et al. [17] found that plots with livestock droppings had less *Sphagnum* cover, suggesting that there could be a negative effect at some stocking levels.

### 4.3 *Sphagnum* frequency in the grazed main experiment and reference plots

The results of the comparison between the grazed main experiment and reference plots, in particular the three times greater *Sphagnum* map frequency in R plots compared to N plots, suggest that the 1954 burns had a negative impact on *Sphagnum* which has persisted for over 60 years. This indicates the importance of considering and critically evaluating ‘control’ treatments when interpreting results from long term experiments. Although the R plots at Hard Hill may have been burned historically prior to the experiment, they are likely to provide a more representative baseline than the N plots. The severity of the 1954 burns is unknown (and could have varied between blocks), and therefore the difference in *Sphagnum* abundance between N and R plots could be a result of combustion, temperature related damage [20], or indirect effects via changes to peat properties [41–43] after the 1954 fires.

The R plots also had more *Sphagnum* than L plots, but a similar amount to S plots, suggesting that the 10-year burning rotation in these plots has mitigated the impact of the initial 1954 burn. The greater hummock height in R plots than in L and N plots (Fig 3) could indicate that hummocks in the R plots are generally older, or have grown at a faster rate over the course of the experiment, whilst the hummock height in S plots (which had no significant difference with any other group) could be due to an intermediate growth rate. NMDS analysis (Fig A in S2 Supplementary Information) showed that R plots were distinct from, and occupied a smaller area of the ordination space than the grazed experimental plots, suggesting that they were more consistent in terms of *Sphagnum* species composition than the S, L and N plots. Apart from burning treatments, the only consistent difference between the main experiment and reference plots is likely to be greater trampling by humans, as the main experiment plots have been surveyed more frequently [44].

### 4.4 *Sphagnum* frequency in past surveys

Analysis of the data from the 1961 survey showed no significant difference in *Sphagnum* abundance between the main experiment treatments at this time (Fig 4). This was expected, as in 1961 all of the main experiment plots had been subject to the same treatment (burned once in 1954). Comparison of the N and R plots in 1965 showed that there was significantly more *Sphagnum* in R plots at this point. This shows that the negative effect of the 1954 burn on *Sphagnum*, observed in the 2015–16 survey, was apparent 11 years after burning.

Although the 1961 and 1965 surveys used the same Domin abundance survey methodology, the *Sphagnum* abundance recorded in the N plots in 1965 was lower than in 1961 (Figs 4 and 5). This could be an artefact of the variation in sample sizes or differing interpretations of the nonlinear Domin scale by surveyors on the two occasions. Alternatively a decrease in *Sphagnum* abundance in N plots between 1961 and 1965 could have been caused by the unusually cold winter of 1962–63 [45], the relatively high levels of atmospheric pollutants such as SO_2_ at this time [36, 37], or an interaction between one of these factors and burning. As R plots were not surveyed in 1961 and S and L plots were not surveyed in 1965, it is not possible to determine whether this difference was specific to the N plots. The difference highlights some of the potential problems with comparing data collected by different surveyors and at different times and makes it difficult to interpret how *Sphagnum* abundance in R plots compared to S and L plots in the early years of the experiment.

### 4.5 Caveats

The Hard Hill experiment has provided a significant amount of published knowledge on prescribed burning impacts [19, 28–30, 34, 46–50]. However, caution is required when extrapolating results to peatland or moorland in general. For example, it has been suggested that local conditions at Moor House including high altitude and high annual rainfall may cause a delay in regeneration of *C*. *vulgaris* compared to other sites [51], which may give other plants, including *Sphagnum*, more opportunity to establish and grow after burning.

The burning carried out for the Hard Hill experiment is likely to have been carefully controlled, and does not represent the full range of burning methods and severities which occur on peatlands. The experiment also represents a relatively small area of a hillslope otherwise dominated by vegetation that has remained unburned for over 90 years, which may influence the hydrology of the plots and provide a source of *Sphagnum* propagules. On sites managed for grouse shooting, a much larger proportion of the site may be burned and fire effects on water availability [22, 43] and the *Sphagnum* propagule bank [30] may be compounded.

## 5. Conclusions

Our results suggest that, as in the case of the 1954 burn, a single fire event can lead to reduced *Sphagnum* over 60 years later. This provides evidence against burning previously unburned (or long-unburned) areas of blanket peatland where *Sphagnum* is present. Thus the recent trend in some parts of the UK for burning encroachment onto areas of peat that have not been burned for at least several decades [9, 15, 16] could reduce *Sphagnum* cover with potentially deleterious impacts on ecosystem function [43, 52]. Shorter rotations may lead to greater *Sphagnum* abundance compared to longer rotations in some cases, though environmental conditions including atmospheric pollution may influence this effect. We would caution against burning on a shorter rotation as a method of encouraging *Sphagnum* because of the potential for other negative effects on peatland function [31, 42, 53]. The longevity and scale of the Hard Hill experiment make it a valuable source of information, but care should be taken when generalising results from any single site, and in particular the specific management history and climate of Moor House should be considered. Furthermore, interpreting historical data can be challenging, particularly where surveyors, methods or sample sizes are inconsistent, and apparent changes over time should be treated with caution.

## Supporting information

S1 Supporting Information *Sphagnum* occurrence in 2015–16. (DOCX)

S2 Supporting Information NMDS analysis of *Sphagnum* species abundance in the grazed experimental treatments and reference plots in 2015–16. (DOCX)
