# Supplementary material for: Impacts of prescribed burning on Sphagnum mosses in a long-term peatland field experiment
Source: PLoS One. 2018 Nov 1;13(11):e0206320. doi: 10.1371/journal.pone.0206320 (PMC6211700; doi:10.1371/journal.pone.0206320)
Supplement: S1 Supporting Information — (DOCX) [file pone.0206320.s001.docx]

**S1 Supporting Information**

***Sphagnum* occurrence in 2015-16**

The transect survey recorded seven *Sphagnum* species in the experiment and reference plots while the mapping survey recorded 12 (Table A). The most common species, representing around 90% of *Sphagnum* recorded, was *S. capillifolium*. Four species (*S. tenellum*, *S. cuspidatum*, *S. russowii* and *S. girgensohnii*) were present in the main experiment plots and absent from the reference plots, while two species (*S. angustifolium* and *S. palustre*) were present in the reference plots and absent from the main experiment plots.

**Table A: Frequencies of *Sphagnum* as a genus and all individual species (names and botanical authorities from the UK Species Inventory) observed in the 2015-16 transect and mapping surveys. One species was recorded for each transect hit, but multiple species could occur in each map square. Experiment (fenced and grazed) n = 24 plots, reference (grazed) n = 4 plots.**

|  |  | Transect hits % | | Map squares % | |
| --- | --- | --- | --- | --- | --- |
| Species | Botanical authority | Experiment | Reference | Experiment | Reference |
| *Sphagnum* spp. | L. | 19.71 | 28.25 | 42.71 | 57.02 |
| *S. capillifolium* | (Ehrh.) Hedw. | 17.50 | 26.50 | 39.57 | 52.53 |
| *S. subnitens* | Russow & Warnst. | 0.88 | 1.00 | 2.91 | 1.74 |
| *S. papillosum* | Lindb. | 0.50 | 0.25 | 1.49 | 0.27 |
| *S. fallax* | (H.Klinggr.) H.Klinggr. | 0.38 | 0.25 | 0.77 | 1.64 |
| *S. angustifolium* | (C.E.O.Jensen ex Russow) C.E.O.Jensen | 0.00 | 0.25 | 0.00 | 2.84 |
| *S. tenellum* | (Brid.) Bory | 0.42 | 0.00 | 0.39 | 0.00 |
| *S. fimbriatum* | Wilson | 0.00 | 0.00 | 0.13 | 0.02 |
| *S. cuspidatum* | Ehrh. ex Hoffm. | 0.00 | 0.00 | 0.05 | 0.00 |
| *S. palustre* | L. | 0.00 | 0.00 | 0.00 | 0.21 |
| *S. magellanicum* | Brid. | 0.00 | 0.00 | 0.02 | 0.14 |
| *S. russowii* | Warnst. | 0.04 | 0.00 | 0.03 | 0.00 |
| *S. girgensohnii* | Russow | 0.00 | 0.00 | 0.02 | 0.00 |
