# Supplementary material for: Impacts of prescribed burning on Sphagnum mosses in a long-term peatland field experiment
Source: PLoS One. 2018 Nov 1;13(11):e0206320. doi: 10.1371/journal.pone.0206320 (PMC6211700; doi:10.1371/journal.pone.0206320)
Supplement: S2 Supporting Information — (DOCX) [file pone.0206320.s002.docx]

**S2 Supporting Information**

**NMDS analysis of *Sphagnum* species abundance in the grazed experimental treatments and reference plots in 2015-16**

Non-metric Multidimensional Scaling (NMDS) ordination analysis was carried out using the map frequency data for all *Sphagnum* species occurring in the grazed experimental plots and reference plots to enable visualisation of how the treatments differed in terms of their overall *Sphagnum* species composition.


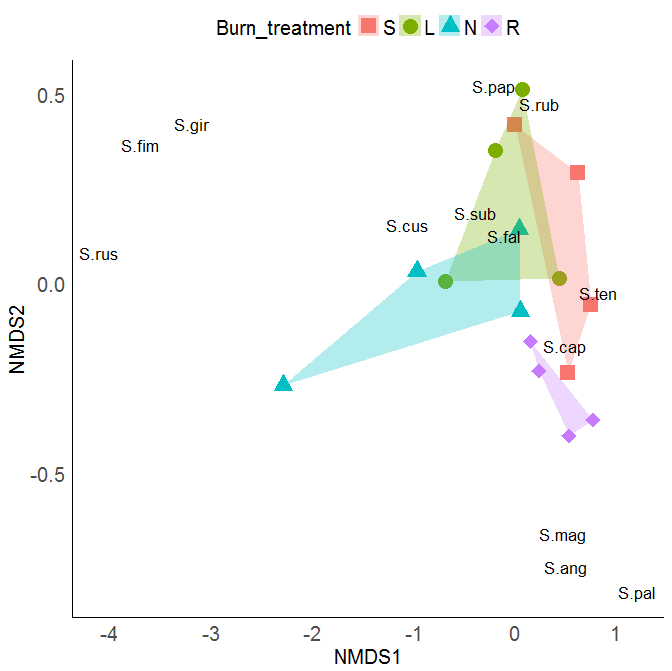


**Fig A: NMDS ordination of *Sphagnum* abundance in the grazed short rotation (S), long rotation (L) no-burn since 1954 (N) and reference plots (R) in 2015-16.**
